# Supplementary figures and images for: Transcriptome Analysis Reveals the Molecular Mechanisms of Carrot Adaptation to Alternaria Leaf Blight
Source: Int J Mol Sci. 2024 Dec 6;25(23):13106. doi: 10.3390/ijms252313106 (PMC11642675; doi:10.3390/ijms252313106)

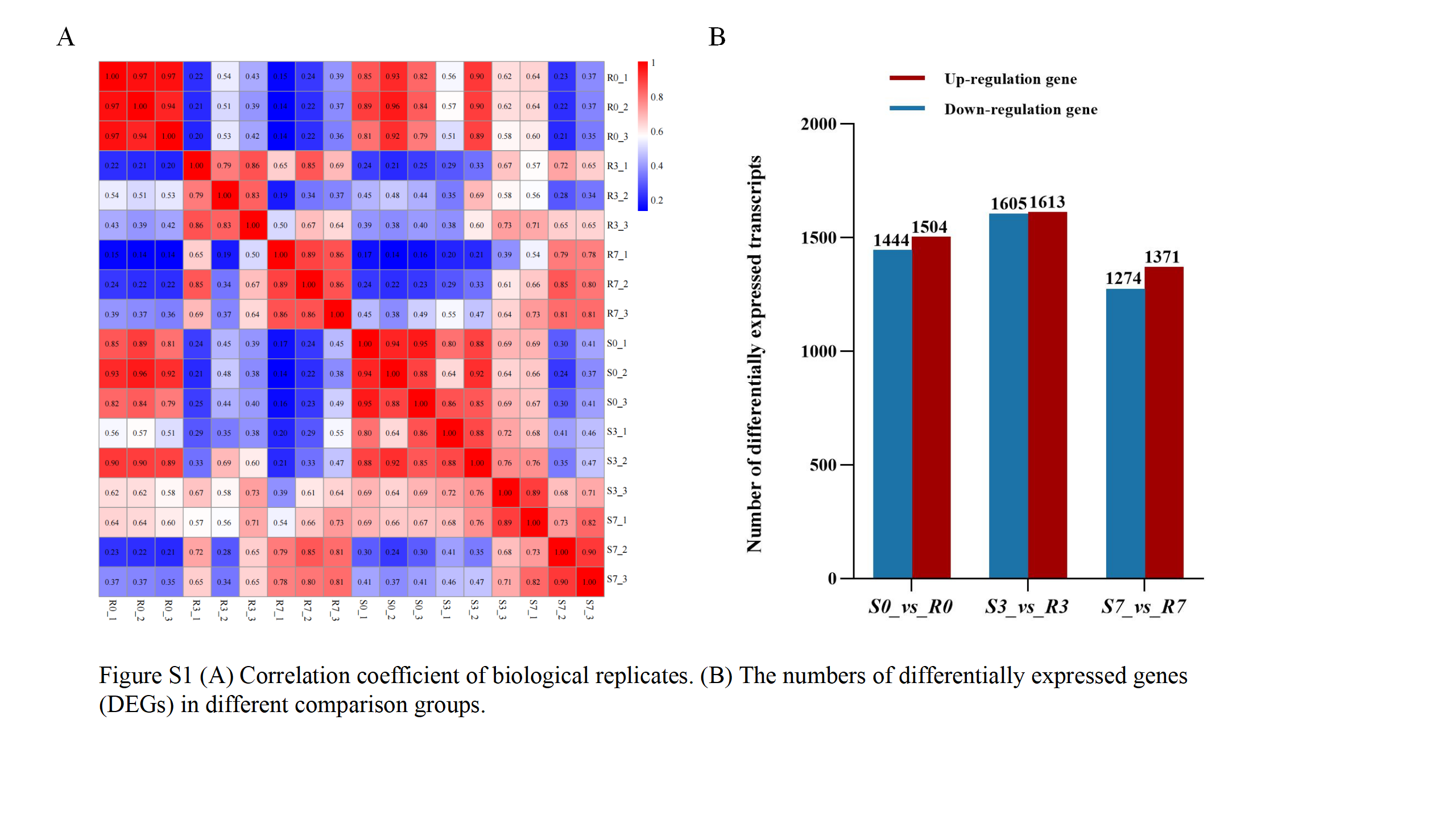

Supplement: Supplementary file 1 [file ijms-25-13106-s001.zip › Figure S1.png]
